# Lambertianic Acid from *Platycladus orientalis* Inhibits Muscle Atrophy in Dexamethasone-Induced C2C12 Muscle Atrophy Cells

**DOI:** 10.3390/plants14091357

**Published:** 2025-04-30

**Authors:** Chan Hee Cho, Si Hyeon Chae, Ngoc Han Le Thi, Sung Hee Um, Seulah Lee, Jae Sik Yu, Ki Sung Kang, Ki Hyun Kim

**Affiliations:** 1 School of Pharmacy, Sungkyunkwan University, Suwon 16419, Republic of Korea; whckswhcks@g.skku.edu (C.H.C.); tlgus3936@naver.com (S.H.C.); 2 College of Korean Medicine, Gachon University, Seongnam 13120, Republic of Korea; ngochanlethi.nhlt@gmail.com; 3 Department of Molecular Cell Biology, School of Medicine, Sungkyunkwan University, Suwon 16419, Republic of Korea; shum@skku.edu; 4 Department of Oriental Medicine Biotechnology, Graduate School of Biotechnology, Kyung Hee University, Yongin 17104, Republic of Korea; lee.seulah@khu.ac.kr; 5 Department of Integrative Biological Sciences and Industry, Sejong University, 209 Neungdong-ro, Gwangjin-gu, Seoul 05006, Republic of Korea; jsyu@sejong.ac.kr

**Keywords:** *Platycladus orientalis*, lambertianic acid, electronic circular dichroism (ECD) calculations, C2C12, muscle atrophy, dexamethasone

## Abstract

*Platycladus orientalis*, an evergreen tree belonging to the Cupressaceae family, has been traditionally used to treat various ailments, including fever, cough, diarrhea, diuresis, cold symptoms, and gastrointestinal disorders in folk medicine. As part of our ongoing investigation aimed at discovering bioactive natural products and elucidating their mechanisms of action from various natural sources, we investigated a methanol (MeOH) extract of *P. orientalis* leaves. This investigation led to the isolation and identification of a labdane-type diterpene, lambertianic acid (LA), via column chromatography and HPLC purification. The structure of LA was elucidated using LC/MS and NMR spectroscopic analyses, including HR-ESIMS, while its absolute configuration was confirmed through electronic circular dichroism (ECD) calculations. Recent studies have reported that labdane-type diterpenes exhibit diverse pharmacological activities, such as anticancer, anti-inflammatory, anti-obesity, and hypolipidemic effects. Notably, LA has been shown to modulate adipocyte metabolism via AMPK signaling; however, its role in skeletal muscle atrophy remains unexplored. Therefore, in this study, we investigated the effects of LA on dexamethasone (Dex)-induced muscle atrophy in C2C12 myotubes. Treatment with LA at concentrations of 25 µM and 50 µM significantly rescued myotube diameter and reduced the expression of atrophy-related proteins, including MuRF-1 and atrogin-1/MAFbx, without compromising cell viability at these moderate concentrations. These findings suggest that LA derived from *P. orientalis* exerts protective effects against skeletal muscle atrophy, highlighting its potential as a promising natural therapeutic candidate for muscle-wasting disorders.

## 1. Introduction

Skeletal muscle atrophy is a pathological condition characterized by a significant reduction in muscle mass, strength, and function, which impairs mobility and increases the risk of various health complications. This condition can be triggered by numerous factors, including aging (sarcopenia), physical inactivity (disuse atrophy), chronic diseases such as cancer and heart failure (cachexia), obesity, and neurodegenerative disorders. The primary biological mechanisms driving muscle atrophy include increased protein degradation and suppressed protein synthesis, primarily regulated through pathways such as the ubiquitin–proteasome system (UPS), the autophagy–lysosome pathway, and key signaling molecules such as forkhead box O (FOXO), nuclear factor kappa-light-chain-enhancer of activated B cells (NF-κB), and myostatin [1]. Inflammatory cytokines such as tumor necrosis factor-alpha (TNF-α) and interleukin-6 (IL-6) further accelerate muscle breakdown by activating catabolic pathways [2]. Moreover, mitochondrial dysfunction, oxidative stress, and impaired neuromuscular signaling contribute to disease progression. Despite the severe consequences of skeletal muscle atrophy, such as reduced quality of life, increased hospitalization rates, and higher mortality, it often goes undiagnosed in its early stages due to the gradual onset of symptoms [3]. Therefore, a deeper understanding of the molecular and cellular mechanisms underlying muscle atrophy is crucial for developing effective preventive measures and therapeutic strategies.

The development of pharmaceuticals for skeletal muscle atrophy is essential due to the limitations of current treatment options, such as exercise therapy, which is often ineffective for patients who are bedridden, elderly, or suffering from severe injuries. Additionally, no FDA-approved drugs specifically target muscle atrophy, highlighting a significant unmet medical need [2]. Promising research, such as the identification of morroniside, which combats inflammation-induced muscle loss by inhibiting NF-κB signaling and promoting protein synthesis, exemplifies the potential of targeted pharmacological interventions [2]. Similarly, research on the transcription factor KLF5 and its inhibitor Am80 has demonstrated potential in preventing muscle degradation due to disuse and corticosteroid exposure [4]. Developing such targeted therapies is vital not only for improving patients’ quality of life but also for reducing the socioeconomic burden associated with muscle-wasting diseases. As skeletal muscle is essential for mobility, metabolic regulation, and overall health, advancing pharmaceutical research and development can provide life-changing solutions for millions of affected individuals. The development of therapeutics for skeletal muscle atrophy derived from natural products holds significant promise due to their diverse bioactive compounds, multi-target effects, and generally favorable safety profiles. Therefore, further research—including bioactivity-guided fractionation and molecular target identification—is essential to harness their therapeutic potential. The discovery of natural product-based therapeutics could offer a sustainable, safe, and effective strategy for combating skeletal muscle atrophy, addressing the current lack of FDA-approved treatments, and ultimately improving patient outcomes.

*Platycladus orientalis* L. [*Thuja orientalis*, *Biota orientalis*] is a species within the Cupressaceae family, characterized as a monoecious and evergreen tree known for its medicinal attributes. It is widely distributed in Korea, China, and Iran [5]. For many years, *P. orientalis* has been used in traditional medicine to treat a variety of conditions, including fever, cough, diarrhea, diuresis, cold symptoms, and gastrointestinal disorders [6]. Previous studies on extracts of this plant have reported various pharmacological properties, including anti-inflammatory, antioxidant, antidiabetic, antimicrobial, anticancer, hair growth-promoting, and neuroprotective activities [7,8,9,10,11,12,13,14]. Recently, *P. orientalis* has garnered medicinal attention as a source of monoterpenes, diterpenes, flavonoids, and lignans. Diterpenes and flavonoids play significant roles in the medicinal attributes associated with the leaves of *P. orientalis* [6,15,16]. One of the most interesting molecules identified in *P. orientalis* is a labdane-type diterpene, which has demonstrated several biological activities, such as anticancer, antibacterial, and antifungal effects [17,18]. Labdane-type diterpenes isolated from the leaves of *P. orientalis* have exhibited antifibrotic activity by modestly reducing the proliferation of HSC-T6 cells in a dose- and time-dependent manner [19]. Additionally, flavonoids derived from *P. orientalis* possess anti-inflammatory effects by inhibiting the biosynthesis of leukotriene B4 (LTB4) and the formation of 5-hydroxyeicosatetraenoic acid (5-HETE) [7].

A search for studies on *P. orientalis* (syn. *T. orientalis*, *B. orientalis*) specifically targeting skeletal muscle atrophy did not yield direct results. However, existing research highlights the therapeutic potential of *P. orientalis* due to its rich composition of bioactive compounds—including flavonoids, lignans, terpenoids, and essential oils—that have demonstrated anti-inflammatory, antioxidant, and cytoprotective properties in various pathological contexts [7]. These mechanisms are closely related to pathways involved in muscle atrophy, such as a reduction in oxidative stress, the inhibition of NF-κB signaling, and the modulation of autophagy and proteasomal degradation. Terpenoids, such as labdane-type diterpenes, and flavonoids, commonly found in *P. orientalis*, are known to inhibit inflammation and oxidative damage—critical contributors to muscle loss in atrophy. Moreover, the essential oils from *P. orientalis* have shown potential in modulating immune responses, which could mitigate the inflammatory cytokine surge associated with muscle degeneration [7]. Given these pharmacological properties, *P. orientalis* holds promise as a source for novel natural therapeutics for skeletal muscle atrophy. Future research should focus on isolating specific active compounds and evaluating their effects in muscle atrophy models.

As part of our ongoing efforts to discover biologically active compounds from diverse natural sources [20,21,22,23,24,25], we collected and prepared methanol (MeOH) extracts of *P. orientalis* leaves for phytochemical investigation, as the MeOH extract demonstrated potential in attenuating muscle atrophy. To isolate a potential bioactive compound from these leaves, we performed column chromatographic separation of the MeOH extract. Subsequent high-performance liquid chromatography (HPLC) purification led to the isolation of a labdane-type diterpene, lambertianic acid (LA), as confirmed by liquid chromatography/mass spectrometry (LC/MS) analysis. The structure of LA was elucidated by combining NMR spectroscopic data with LC/MS analysis results, and its absolute configuration was determined. LA was subsequently tested for its effects on inhibiting muscle atrophy in C2C12 cells, which are derived from mouse skeletal muscle myoblasts. Here, we describe the isolation and structural characterization of LA, including the determination of its absolute configuration, as well as its potential effects on the regulation of muscle atrophy.

## 2. Results

### 2.1. Isolation and Structure Elucidation of Lambertianic Acid

**Figure 1:**
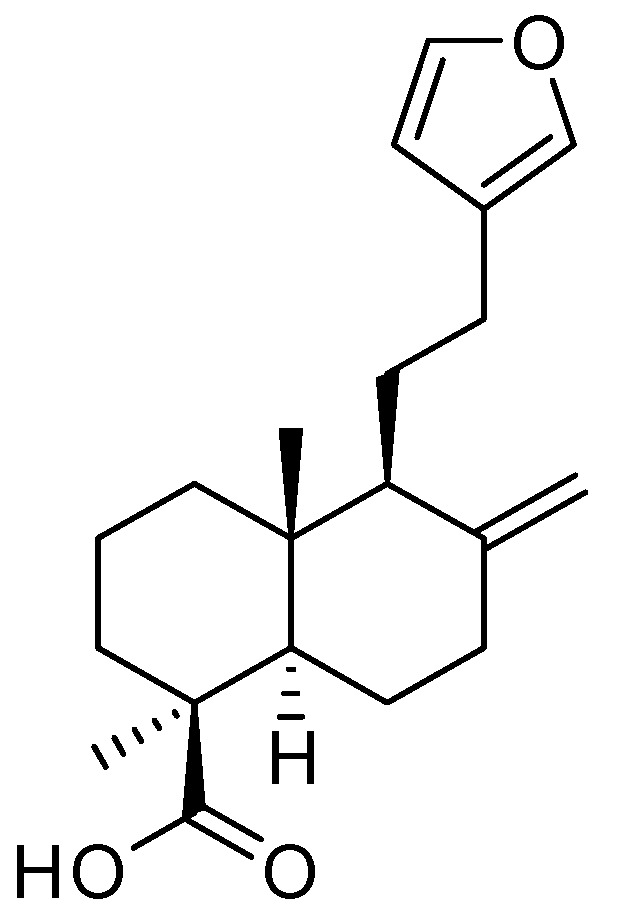
Chemical structure of lambertianic acid.

The dried leaves of *P. orientalis* were extracted with 80% methanol (MeOH), and treatment with 50 μg/mL of the MeOH extract in dexamethasone (Dex)-induced atrophic cells significantly improved myotube diameter compared to the Dex-only group, without affecting cell viability (Figure S1). These results suggest that the extract has the potential to attenuate muscle atrophy. The MeOH extract was subsequently partitioned using solvents of varying polarity. Among the resulting fractions, the *n*-hexane fraction—identified by LC-MS analysis as being enriched in labdane-type diterpenes—was selected for further isolation. Chromatographic techniques, including open column chromatography, preparative HPLC, and semi-preparative HPLC, were employed to isolate a labdane-type diterpene. The structure of the isolated compound was confirmed as lambertianic acid (LA) (Figure 1) by comparing its NMR spectroscopic data and LC-MS analysis (Figures S2 and S3) with previously reported values [26].

**Figure 2:**
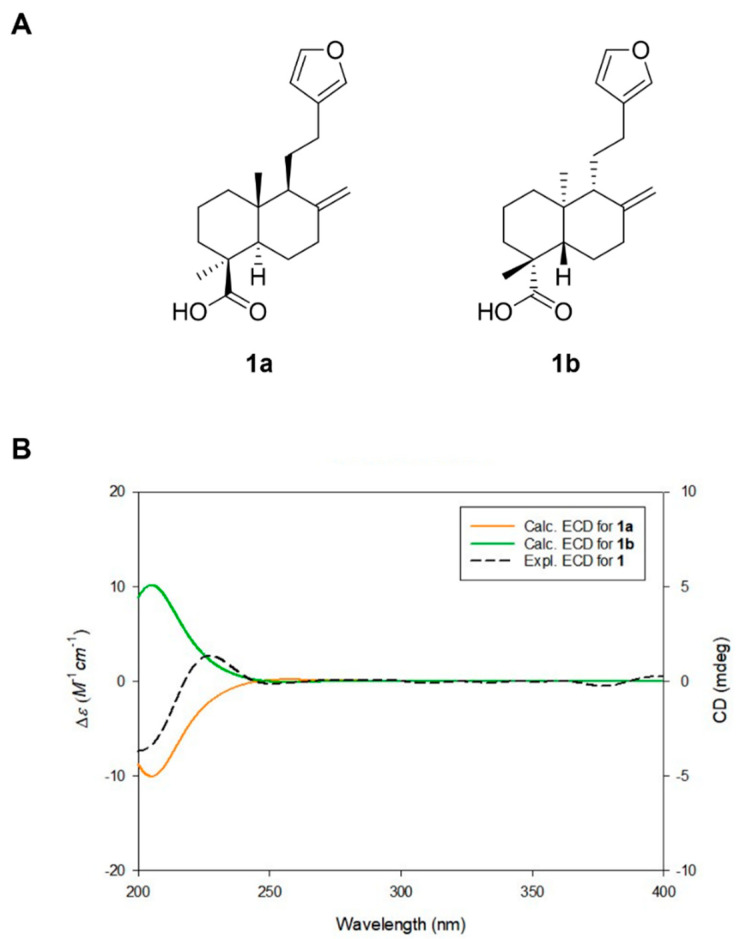
(**A**) Two possible isomers of LA for ECD calculations. (**B**) Experimental and calculated ECD spectra of LA.

To confirm the absolute configuration of the lambertianic acid (LA) isolated in this study, quantum chemical calculations for ECD simulations were performed. ECD data were simulated for two isomers—**1a** (4*S*,5*R*,9*S*,20*R*) and **1b** (4*R*,5*S*,9*R*,10*S*)—using density functional theory (DFT)-based calculations. The calculated ECD data were then compared with the experimental ECD spectrum of LA (Figure 2). The Boltzmann-averaged computed ECD spectrum of **1a** (4*S*,5*R*,9*S*,10*R*) matched well with the experimental spectrum, indicating that the absolute configuration of LA is 4*S*,5*R*,9*S*,10*R*. Accordingly, the chemical structure of LA, including its absolute configuration, was determined (Figure 2).

### 2.2. Effect of LA and Dexamethasone on C2C12 Cell Viability

**Figure 3:**
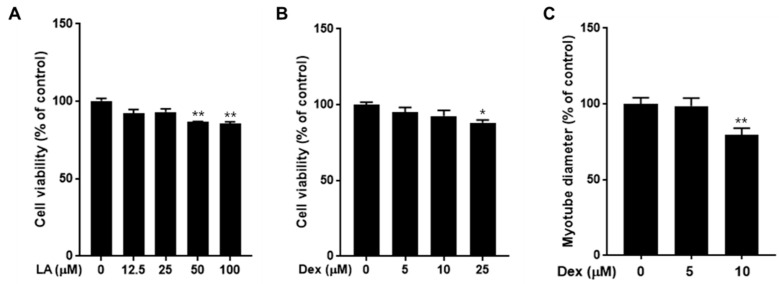
Effects of LA and dexamethasone (Dex) on cell viability in C2C12 and quantitative image analysis of myotube. C2C12 cells (1 × 10^4^ cells/well, 96-well plates) were treated with LA (**A**) or Dex (**B**) for 24 h. Cell viability was measured using an EZ-cytox cell viability solution. (**C**) C2C12-differentiated myotubes were treated with Dex for 48 h. Average diameters of at least 25 myotubes were determined for each condition at three points (*n* = 3 measurements/myotube). The data are expressed as mean ± SEM of triplicate experiments. * *p* < 0.05; ** *p* < 0.01 vs. the control group.

Before investigating the potential effects of LA on the regulation of muscle atrophy, we first assessed the cytotoxicity of both LA and dexamethasone (Dex) on C2C12 muscle cells. As shown in Figure 3A, treatment with LA at concentrations of 12.5 and 25 μM did not affect cell viability, whereas higher concentrations induced cell death. Specifically, LA at concentrations of 50 and 100 μM significantly decreased cell viability by 13.5% and 14.4%, respectively. Similarly, treatment with 25 μM Dex resulted in significant cell death (Figure 3B). Next, we examined the diameter of myotubes after treatment with 5 μM and 10 μM concentrations of Dex. Following treatment, C2C12 cells showed a significant decrease in myotube diameter in the 10 μM Dex group (Figure 3C). Consequently, 10 μM Dex was used for subsequent experiments.

### 2.3. Effect of LA on C2C12 Myotubes Fiber Diameter

**Figure 4:**
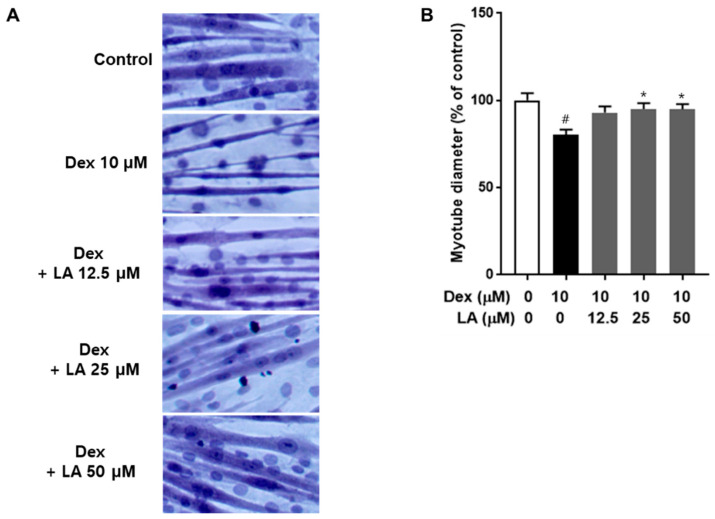
Effect of LA on dexamethasone-induced atrophy in C2C12 myotubes. (**A**) Differentiated C2C12 myotubes were treated with Dex alone or co-treated with various concentrations of LA for 48 h. Representative images of hematoxylin and eosin staining are shown for the control, Dex, and Dex + LA treatments. (**B**) The average diameters of at least 25 myotubes were measured at three different points for each condition (*n* = 3 measurements/myotube). Data are expressed as mean ± SEM of triplicate experiments. # *p* < 0.05 vs. the control group; * *p* < 0.05 vs. the Dex-induced group.

We assessed whether LA could rescue C2C12 myotubes from Dex-induced atrophy by treating the cells with various concentrations of LA in the presence of 10 μM Dex. Our results indicate that LA has the potential to attenuate muscle atrophy. Specifically, co-treatment with 25 μM and 50 μM LA improved myotube diameter by 14.9% and 14.8%, respectively, compared to the Dex-only group (Figure 4).

### 2.4. Effect of LA on Atrogin-1 and MuRF-1 Expression in Dex-Treated C2C12 Myotubes

**Figure 5:**
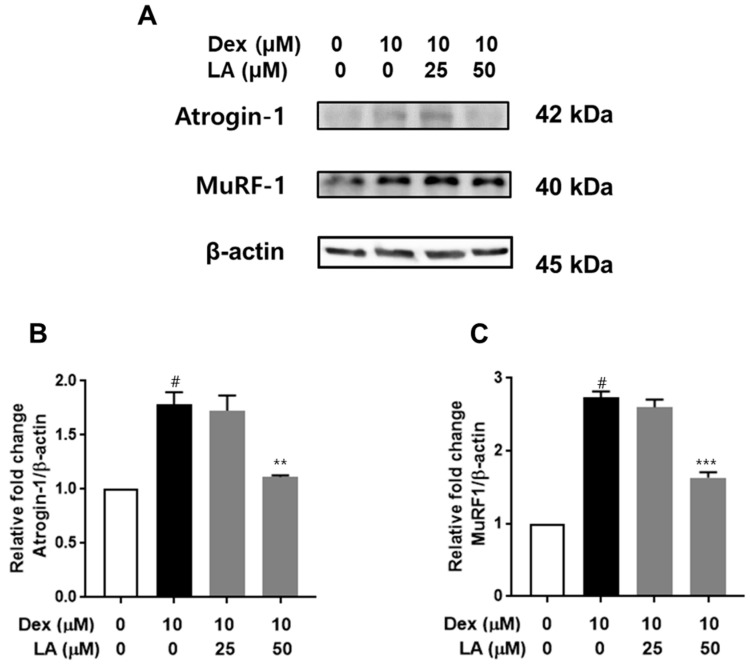
Effect of LA on Atrogin-1 and MuRF-1 expression in Dex-treated C2C12 myotubes. (**A**) Representative Western blot images showing protein expression levels of Atrogin-1 and MuRF-1. (**B**) Quantitative analysis of Atrogin-1 protein expression. (**C**) Quantitative analysis of MuRF-1 protein expression. C2C12 myotubes were treated with LA in the presence or absence of 10 μM Dex for 48 h. Data are expressed as mean ± SEM from duplicate experiments. # *p* < 0.05 vs. the control group, ** *p* < 0.01; *** *p* < 0.001 vs. the Dex-induced group.

To evaluate the effects of LA on Atrogin-1 and MuRF-1 expression [27], we performed Western blot analyses. Dex treatment resulted in a 1.7-fold increase in Atrogin-1 protein levels and an approximate 2.7-fold increase in MuRF-1 levels compared to the control (Figure 5). Importantly, co-treatment with 50 μM LA significantly downregulated the expression of these proteins, reducing Atrogin-1 and MuRF-1 levels to 1.11-fold and 1.63-fold of the control, respectively.

## 3. Discussion

Lambertianic acid (LA) is a labdane-type diterpene first identified from *Pinus lambertiana* and is an optical isomer of the natural compound daniellic acid [26,28]. LA exhibits a range of pharmacological activities, including anticancer, anti-allergic, and anti-obesity effects, and it has been shown to inhibit apoptotic processes in breast, liver, lung, and prostate cancers [29,30,31,32]. In addition, LA isolated from *Platycladus orientalis* has demonstrated anti-allergic and anti-inflammatory responses in bone-marrow-derived mast cells [33]. Moreover, an LA-enriched ethanol extract of *Pinus koraiensis* was found to reduce fat concentration and intracellular triglyceride levels in 3T3-L1 cells by suppressing the expression of PPAR-γ, C/EBPα, adiponectin, and SREBP-1, as well as modulating the AMPK signaling pathway [34]. A previous study has identified that crosstalk between adipocytes and skeletal muscle contributes to protein degradation and muscle atrophy [35]. Based on this, we hypothesized that inhibiting adipogenesis may reduce protein degradation in muscle cells. Furthermore, other studies have shown that increased AMPK expression promotes adipocyte degradation and suppresses protein degradation in muscle cells [36]. These findings suggest that LA might serve as a potential treatment for skeletal muscle atrophy—a possibility that has not been explored to date.

In this study, we evaluated the inhibitory effects of LA against dexamethasone (Dex)-induced muscle atrophy in C2C12 myotubes. Dex, a synthetic glucocorticoid analog, is commonly used to model muscle atrophy, as it induces the expression of atrophy-related genes, including Muscle RING-finger 1 (MuRF-1) and muscle atrophy F-box (MAFbx, also known as atrogin-1) [37,38]. Muscle RING finger 1 (MuRF-1) and muscle atrophy F box (MAFbx, also known as atrogin 1) are E3 ubiquitin ligases that play crucial roles in regulating protein degradation during skeletal muscle atrophy. They are key components of the ubiquitin proteasome system (UPS), the primary pathway for protein catabolism in muscle wasting conditions [27]. These proteins are upregulated early in the atrophy process, preceding the loss of muscle mass [37,38]. We employed 10 µM Dex to establish an in vitro muscle atrophy model, as described previously [39,40]. Our results showed that, except at high concentrations (50 and 100 μM), LA did not adversely affect cell viability. Notably, co-treatment with LA at concentrations of 25 μM and 50 μM significantly rescued myotube diameter and attenuated the Dex-induced upregulation of MuRF-1 and atrogin-1/MAFbx protein expression (Figure 5). These findings suggest that LA holds significant therapeutic potential for preventing and treating skeletal muscle atrophy.

Labdane-type diterpenes, a class of naturally occurring diterpenoids present in various medicinal plants, are renowned for their potent anti-inflammatory, antioxidant, and cytoprotective properties—attributes that are critical for modulating the molecular pathways involved in muscle atrophy. For instance, key labdane-type diterpenes such as andrographolide (from *Andrographis paniculata*), forskolin (from *Coleus forskohlii*), and oridonin (from *Rabdosia rubescens*) have shown promise in modulating signaling pathways like NF-κB, PI3K/Akt, and FOXO, which are essential in maintaining the balance between muscle protein synthesis and degradation. Andrographolide, for example, inhibits the NF-κB pathway, thereby reducing the production of inflammatory cytokines that contribute to muscle wasting. Forskolin activates adenylyl cyclase, increases intracellular cAMP levels, and promotes Akt activation, which, in turn, suppresses the expression of MuRF-1 and MAFbx (atrogin-1), key markers of muscle protein degradation. Similarly, oridonin exhibits anti-apoptotic and anti-inflammatory effects that may help mitigate muscle cell damage during atrophy. However, to date, there have been no direct studies linking labdane-type diterpenes with the prevention of skeletal muscle atrophy. Thus, our study provides the first experimental evidence demonstrating that LA, as a labdane-type diterpene, has a protective effect against skeletal muscle atrophy, highlighting its promise as a potential therapeutic phytochemical.

## 4. Materials and Methods

### 4.1. General Experimental Procedures

The optical rotations were obtained using a Jasco P-1020 polarimeter (Jasco, Easton, MD, USA). Electronic circular dichroism (ECD) spectra were measured using a Jasco J-1500 spectropolarimeter (Jasco). Nuclear magnetic resonance (NMR) spectra were recorded on a Bruker AVANCE III HD 850 NMR spectrometer at 850 MHz for ^1^H and 212.5 MHz for ^13^C, with chemical shifts reported in parts per million (ppm, δ). Preparative high-performance liquid chromatography (HPLC) was performed using a Waters 1525 Binary HPLC pump coupled with a Waters 996 Photodiode Array Detector (Waters Corporation, Milford, CT, USA), utilizing a Hector C_18_ column (250 × 21.2 mm^2^, 5 µm; flow rate: 5 mL/min; Rstech Corporation, Daejeon, Republic of Korea). Semi-preparative HPLC was carried out on an Agilent 1200 Series system equipped with a G1311A quaternary pump and diode array detector, utilizing a Phenomenex Luna C_18_ column (250 × 10 mm, 10 µm; flow rate: 2 mL/min; Phenomenex, Torrance, CA, USA). High-resolution electrospray ionization mass spectrometry (HR-ESI-MS) data were acquired using an Agilent 6545 Q-TOF LC/MS spectrometer with an EclipsePlus C_18_ 95 Å column (50 × 2.1 mm, 1.8 µm; flow rate: 0.3 mL/min; Agilent Technologies). LC/MS analysis was performed on an Agilent 1200 Series HPLC system (Agilent Technologies, Santa Clara, CA, USA) equipped with a diode array detector and a 6130 Series ESI mass spectrometer, utilizing an analytical Kinetex C_18_ 100 Å column (100 mm × 2.1 mm i.d., 5 µm; Phenomenex, Torrance, CA, USA). Finally, Precoated silica gel F_254_ plates and RP-C_18_ F_254_s plates (Merck, Darmstadt, Germany) were used for thin-layer chromatography (TLC). Spots were detected under UV light or by heating after spraying with anisaldehyde–sulfuric acid.

### 4.2. Plant Material

The leaves of *P. orientalis* were collected in March 2023 from Yeongcheon-si, Gyeongsangbuk-do province, Republic of Korea. The plant material was authenticated by Prof. K. H. Kim, one of the authors of this paper. A voucher specimen (CB-2023) has been deposited in the herbarium of the School of Pharmacy at Sungkyunkwan University, Suwon, Republic of Korea.

### 4.3. Extraction and Isolation

Dried whole leaves of *P. orientalis* (3.1 kg) were extracted by sonicating three times (12.0 L × 3) for 90 min in 80% MeOH at room temperature, followed by filtration. The combined filtrate was then evaporated in vacuo to yield a crude MeOH extract (559.8 g). This extract was suspended in distilled water (700 mL) and partitioned successively with *n*-hexane, dichloromethane (CH_2_Cl_2_), ethyl acetate (EtOAc), and *n*-butanol (n-BuOH) (each 700 mL × 3), affording four fractions: *n*-hexane-soluble (67.4 g), CH_2_Cl_2_-soluble (62.4 g), EtOAc-soluble (38.4 g), and *n*-BuOH-soluble (87.6 g). The *n*-hexane-soluble fraction (31.2 g) was subjected to silica gel column chromatography using a gradient solvent system of CH_2_Cl_2_/MeOH (10:1 to 1:1), yielding 15 fractions (fr. 1–fr. 15). Fraction 7 (5.6 g) was further separated by preparative reversed-phase HPLC (Hector C_18_, 250 × 21.2 mm^2^ i.d., 5 μm) using a gradient system of MeOH/H_2_O (8:2 to 1:0, *v*/*v*; flow rate: 5 mL/min), which provided ten subfractions (fr. 7-1 to fr. 7-10). Among these, LA (7.2 mg) was isolated from subfraction fr. 7-6 via semi-preparative reversed-phase HPLC (Phenomenex C_18_ 100 Å column, 250 × 2.1 mm i.d., 10 μm) using an isocratic system of 90% MeCN/H_2_O (flow rate: 2 mL/min).

#### Lambertianic Acid (LA)

Colorless oil; ${\left[\alpha \right]}_{D}^{25}$ +53 (*c* 0.36, MeOH); UV (MeOH) λ_max_ (log ε) 220 (3.62); ECD (MeOH) λ_max_ (Δε) 205 (−0.75), 220 (0.48) nm; ESI-MS *m*/*z*: 317.2 [M + H]^+^; HR-ESIMS (positive ion mode) *m/z* 317.2122 [M + H]^+^ (calcd. for C_20_H_29_O_3_, 317.2117); NMR data as reported [26].

### 4.4. DFT Based ECD Calculations of LA

Initial conformational searches for LA were conducted using the MMFF94 force field in the MacroModel (version 2021-4, Schrödinger LLC, Broadway, NY, USA) program. A mixed torsional/low-mode sampling method was employed in the gas phase with a 20 kJ/mol energy window and a maximum of 10,000 iterations. Conformer minimization was carried out using the Polak–Ribiere conjugate gradient (PRCG) algorithm, with a convergence threshold set at 0.001 kJ·(mol·Å)^–1^ based on the root-mean-square gradient over a maximum of 10,000 iterations. Conformers found within 5 kJ/mol in the MMFF force field were differentiated and subsequently selected for geometry optimization using TmoleX 4.3.2 under density functional theory settings at the B3-LYP/6-31+G(d,p) level [41].

ECD calculations for the **1a** and **1b** conformers were performed at the B3LYP/6-31+G(d,p) level of theory. The calculated ECD spectra were generated by overlaying each transition, where *σ* represents the width of the band at a height of 1/e (as described in Equation (1)), and Δ*E_i_* and *R_i_* denote the excitation energy and rotatory strength of transition *i*, respectively [42]. In this study, *σ* was set to 0.20 eV. The excitation energies and rotational strengths for the ECD spectra were calculated based on the Boltzmann populations of the conformers. ECD visualization was performed using SigmaPlot 14.0.

(1) $$∆\epsilon \left(E\right)=\frac{1}{2.297\times 10^{-39}}\frac{1}{\sqrt{2\pi \sigma }}\sum_{A}^{i}∆E_{i}R_{i}e^{\left[-{\left(E-∆E_{i}\right)}^{2}/{\left(2\sigma \right)}^{2}\right]}$$

### 4.5. Cell Culture

C2C12 myoblasts were cultured in DMEM supplemented with 10% FBS at 37 °C in a 5% CO_2_ atmosphere. To induce differentiation, once the cells reached 90% confluence, the medium was switched to DMEM containing 2% horse serum (HS), and the cells were maintained under these conditions until fully differentiated into multinucleated, elongated myotubes [43]. The medium was replaced every two days during the five- to six-day differentiation process. For the treatment studies, fully differentiated C2C12 myotubes were then treated with 10 μM dexamethasone (Dex) to induce atrophy, with or without the test compound, for 48 h.

### 4.6. Cell Viability

C2C12 cells were seeded onto 96-well plates at a density of 10^4^ cells per well and starved for 24 h. Subsequently, the cells were treated with the compound at concentrations of 12.5, 25, 50, or 100 μM, or with dexamethasone (Dex) at 5, 10, or 25 μM, each applied individually. After 24 h of incubation, cell viability was measured using an Ez-Cytox kit (DoGenBio, Seoul, Republic of Korea) [44].

### 4.7. Muscle Atrophy Determination

Myotube diameters were measured via hematoxylin–eosin staining, as described by Cardiff, R. D. et al. (2014) [45], with modifications. After treatment, myotubes were washed with Dulbecco’s Phosphate-Buffered Saline (DPBS) and fixed with 4% formaldehyde for 30 min. The cells were then rinsed once with DPBS, followed by dehydration with 100%, 90%, 80%, and 70% ethanol for 3 min each. Subsequently, cells were stained with hematoxylin for 5 min and rinsed with tap water for 5 min. Eosin Y staining was performed for 1 min, and excess dye was removed by sequential exposure to 70%, 80%, and 90% ethanol for 2 min each. Micrographs were captured at 100× and 200× magnifications in three randomly selected areas. Myotube diameters were quantified using ImageJ software, version 1.52a (National Institutes of Health, Bethesda, MD, USA).

### 4.8. Western Blot

C2C12 cells were seeded in 6-well plates, grown to 90% confluence, and differentiated into myotubes over 5 days. Subsequently, the myotubes were treated with various concentrations of LA for 24 h. Following treatment, cell extracts were prepared using radioimmunoprecipitation assay (RIPA) buffer (Cell Signaling, Danvers, MA, USA) supplemented with a 1 × protease inhibitor cocktail and 1 mM phenylmethylsulfonyl fluoride (PMSF). Protein samples were separated on polyacrylamide gels and transferred to polyvinylidene fluoride (PVDF) membranes [46]. These membranes were blocked with 5% skim milk in Tris-buffered saline containing 0.001% Tween 20 and incubated with primary antibodies overnight at 4 °C. The membranes were then incubated with horseradish peroxidase (HRP)-conjugated secondary antibodies at room temperature for 1 h. Immunoreactivity was detected using enhanced chemiluminescence (ECL) reagents (GE Healthcare Biosciences, Waltham, MA, USA). Protein band intensities were quantified using ImageJ software (NIH, Bethesda, MD, USA). Anti β actin (#4970) was obtained from Cell Signaling Technology (Danvers, MA, USA), anti-Atrogin-1 (sc-166806) was obtained from Santa Cruz Biotechnology (Dallas, TX, USA), and anti-MuRF1 (55456-1-AP) from Proteintech (Rosemont, IL, USA). The β actin antibody served as the internal control, and protein quantities were normalized to the intensity of the internal control bands [47].

### 4.9. Statistical Analyses

All data are presented as the mean ± standard error of the mean (SEM) from at least three independent experiments. Statistical significance was determined using Student’s *t*-tests and one-way analysis of variance (ANOVA) with GraphPad Prism 7.0 (GraphPad Software Inc., La Jolla, CA, USA), followed by Tukey’s post hoc test. A *p*-value of less than 0.05 was considered statistically significant.

## 5. Conclusions

The phytochemical investigation of the MeOH extract from *P. orientalis* leaves, aimed at discovering bioactive natural products with an effect against skeletal muscle atrophy, led to the isolation and identification of a labdane-type diterpene, lambertianic acid (LA), via column chromatography and HPLC purification. The structure of LA was elucidated using LC/MS and NMR spectroscopic analyses, supplemented by computational methods for ECD calculations. Our study further evaluated the effects of LA on dexamethasone (Dex)-induced muscle atrophy in C2C12 myotubes, revealing that LA alleviates Dex-induced atrophy without compromising cell viability and significantly attenuates the protein expression of MuRF-1 and Atrogin-1, markers that are upregulated early in the atrophy process. These findings suggest that LA may be developed as a potential agent for the treatment of Dex-induced muscle atrophy, highlighting its promise as a natural therapeutic candidate for muscle-wasting disorders. However, the efficacy of LA has, thus far, been limited to Dex-induced atrophy models, and its therapeutic potential should be further evaluated in other models of muscle atrophy as well as in in vivo studies.

## Data Availability

The original contributions presented in this study are included in the article/Supplementary Material. Further inquiries can be directed to the corresponding authors.

## Supplementary Materials

The following supporting information can be downloaded at: https://www.mdpi.com/article/10.3390/plants14091357/s1, Figure S1: Effects of MeOH extract of *P. orientalis* leaves on dexamethasone-induced atrophy in C2C12 myotubes; Figure S2: The HR-ESI-MS data (positive-ion mode) of **1**; Figure S3: The ^1^H NMR spectrum of **1** (CD_3_OD, 850 MHz).
