# Supplementary material for: Lambertianic Acid from Platycladus orientalis Inhibits Muscle Atrophy in Dexamethasone-Induced C2C12 Muscle Atrophy Cells
Source: Plants (Basel). 2025 Apr 30;14(9):1357. doi: 10.3390/plants14091357 (PMC12073373; doi:10.3390/plants14091357)

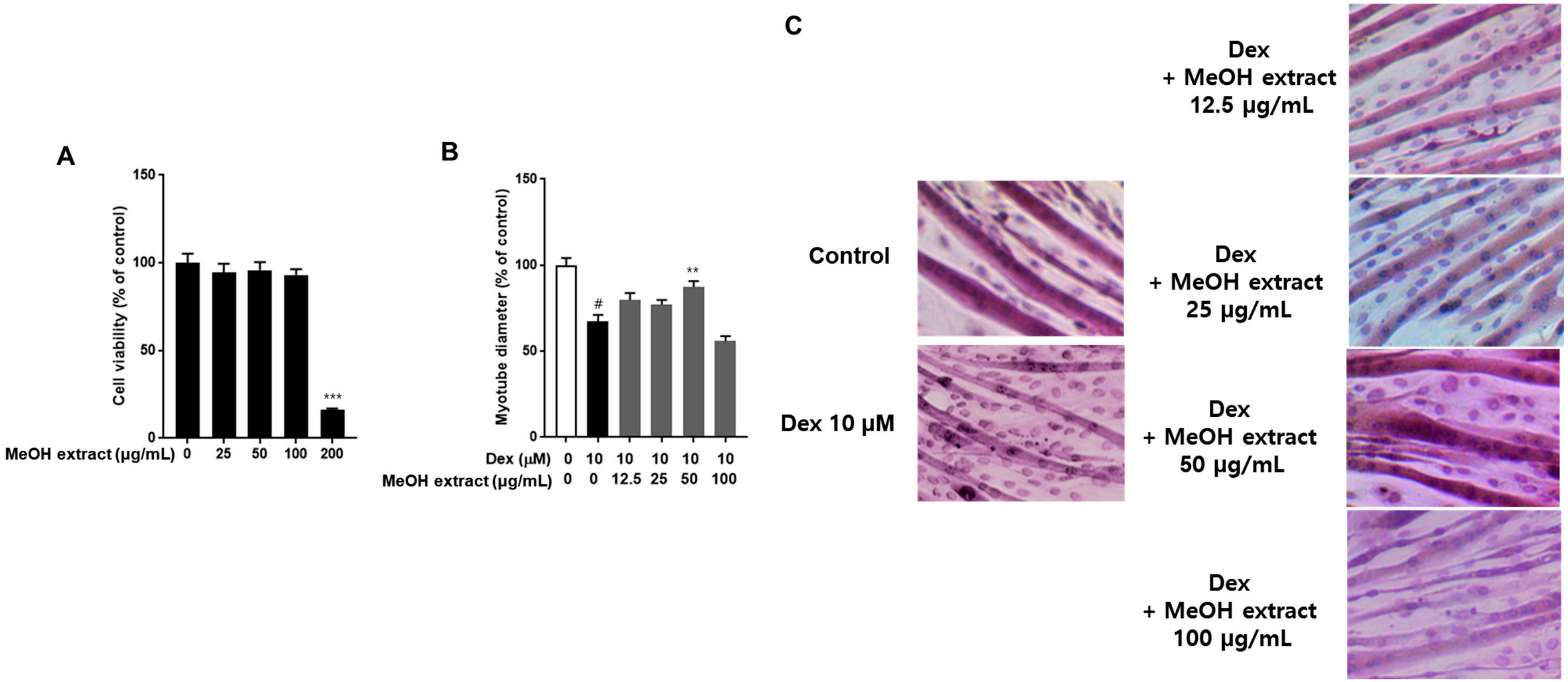

Figure S1. Effects of MeOH extract of *P. orientalis* leaves on dexamethasone-induced atrophy in C2C12 myotubes. (A) C2C12 cells (1×10^4 cells/well, 96-well plates) were treated with MeOH extract for 24 h. Cell viability was measured using an EZ-cytox cell viability solution. The data are expressed as mean ± SEM of triplicate experiments. *** $p < 0.001$ vs. the control group (B) The average diameters of at least 25 myotubes were measured at three different points for each condition (n = 3 measurements/myotube). (C) Differentiated C2C12 myotubes were treated with Dex alone or co-treated with various concentrations of MeOH extract for 48 h. Representative images of hematoxylin and eosin staining are shown for the control, Dex, and Dex + MeOH extract treatments. Data are expressed as mean ± SEM of triplicate experiments. #$p < 0.05$ vs. the control group; **$p < 0.01$ vs. the Dex-induced group.

Figure S2. The HR-ESIMS (positive ion mode) data of **1**

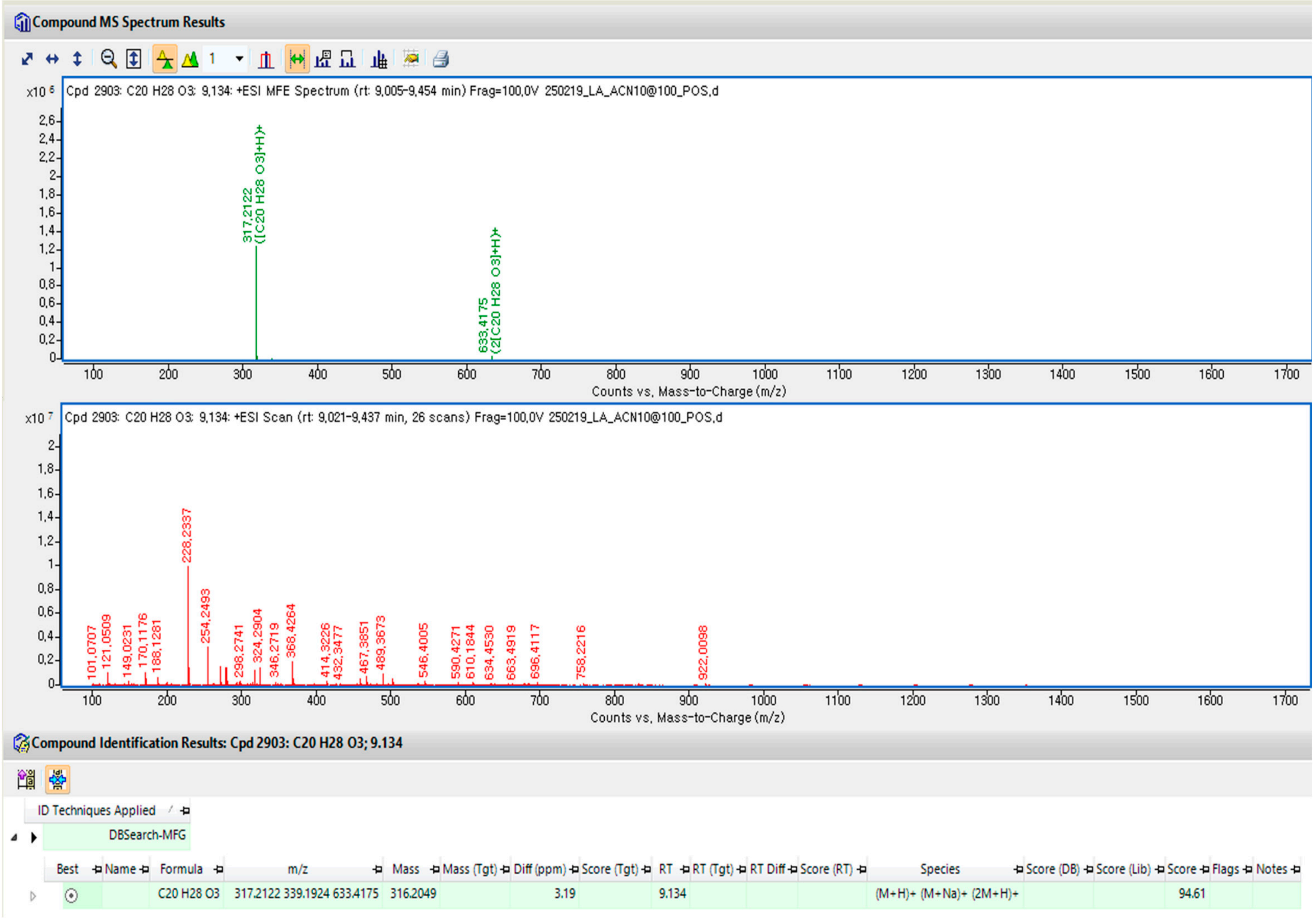

Figure S3. The ^{1}H NMR spectrum of **1** (CD_3OD, 850 MHz)

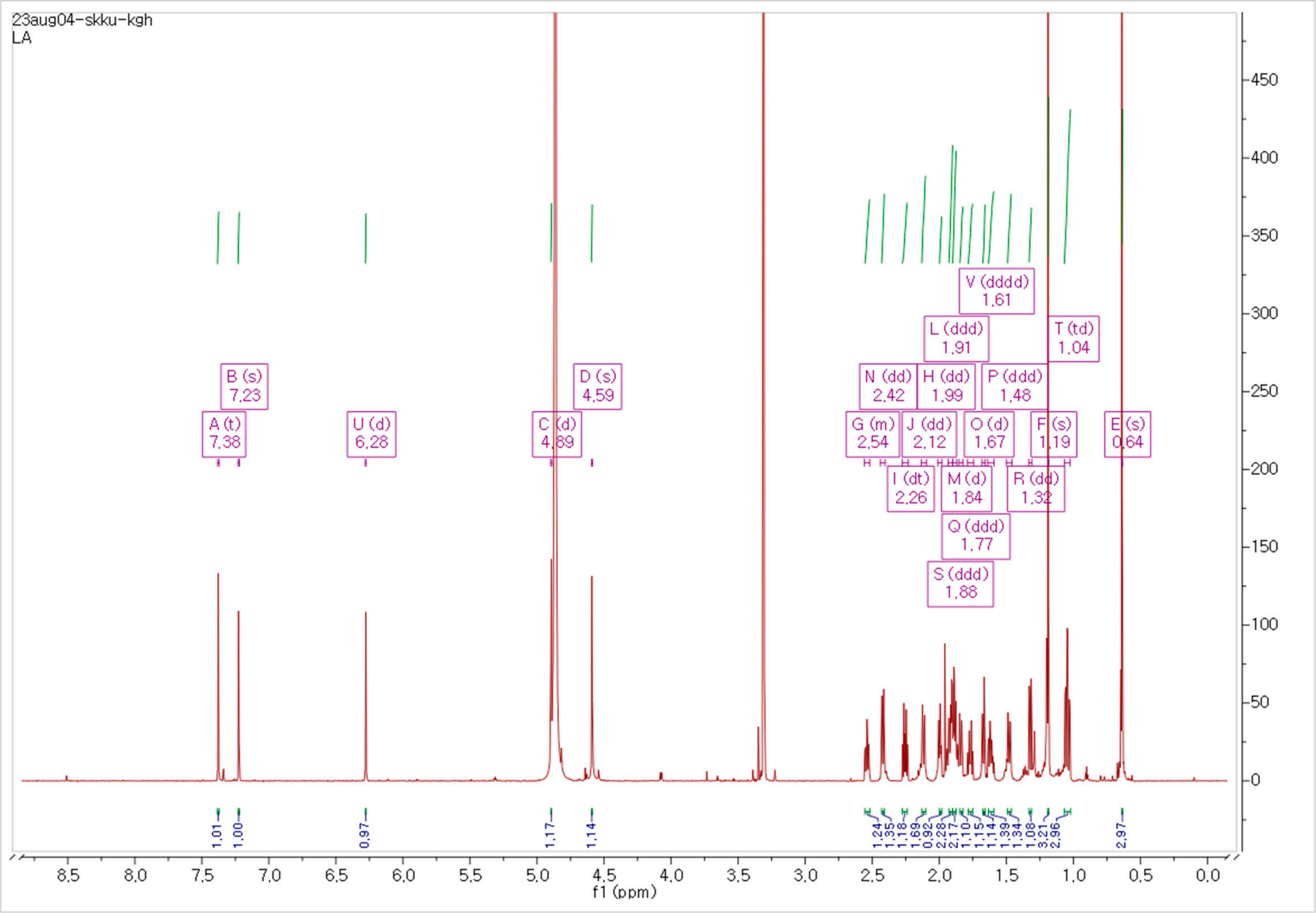

Supplement: Supplementary file 1 [file plants-14-01357-s001.zip › plants-3523425-supplementary.pdf]
